# Evaluation of the RAS signaling network in response to MEK inhibition using organoids derived from a familial adenomatous polyposis patient

**DOI:** 10.1038/s41598-020-74530-x

**Published:** 2020-10-15

**Authors:** Hiroki Osumi, Atsushi Muroi, Mizuho Sakahara, Hiroshi Kawachi, Takuya Okamoto, Yasuko Natsume, Hitomi Yamanaka, Hiroshi Takano, Daisuke Kusama, Eiji Shinozaki, Akira Ooki, Kensei Yamaguchi, Masashi Ueno, Kengo Takeuchi, Tetsuo Noda, Satoshi Nagayama, Naohiko Koshikawa, Ryoji Yao

**Affiliations:** 1 Department of Cell Biology, Cancer Institute, Japanese Foundation for Cancer Research, 3-8-31 Ariake, Koto-ku, Tokyo, 135-8550 Japan; 2 Department of Gastroenterology, Cancer Institute Hospital, Japanese Foundation for Cancer Research, 3-8-31 Ariake, Koto-ku, Tokyo, 135-8550 Japan; 3 Director’s Office, Cancer Institute, Japanese Foundation for Cancer Research, 3-8-31 Ariake, Koto-ku, Tokyo, 135-8550 Japan; 4 Division of Cancer Cell Research, Kanagawa Cancer Center Research Institute, 2-3-2 Nakao, Yokohama, Kanagawa 241-8515 Japan; 5 Division of Pathology, Cancer Institute Hospital, Department of Pathology, Cancer Institute, Japanese Foundation for Cancer Research, 3-8-31 Ariake, Koto-ku, Tokyo, 135-8550 Japan; 6 Department of Gastroenterological Surgery, Cancer Institute Hospital, Japanese Foundation for Cancer Research, 3-8-31 Ariake, Koto-ku, Tokyo, 135-8550 Japan

**Keywords:** Gastrointestinal cancer, Cell signalling

## Abstract

RAS signaling is a promising target for colorectal cancer (CRC) therapy, and a variety of selective inhibitors have been developed. However, their use has often failed to demonstrate a significant benefit in CRC patients. Here, we used patient-derived organoids (PDOs) derived from a familial adenomatous polyposis (FAP) patient to analyze the response to chemotherapeutic agents targeting EGFR, BRAF and MEK. We found that PDOs carrying KRAS mutations were resistant to MEK inhibition, while those harboring the BRAF class 3 mutation were hypersensitive. We used a systematic approach to examine the phosphorylation of RAS effectors using reverse-phase protein array (RPPA) and found increased phosphorylation of MEK induced by binimetinib. A high basal level of ERK phosphorylation and its rebound activation after MEK inhibition were detected in KRAS-mutant PDOs. Notably, the phosphorylation of EGFR and AKT was more closely correlated with that of MEK than that of ERK. Transcriptome analysis identified MYC-mediated transcription and IFN signaling as significantly correlated gene sets in MEK inhibition. Our experiments demonstrated that RPPA analysis of PDOs, in combination with the genome and transcriptome, is a useful preclinical research platform to understand RAS signaling and provides clues for the development of chemotherapeutic strategies.

## Introduction

Activating mutations in the RAS and BRAF proteins are found in approximately 50%^1^ and 10%^2,3^, respectively, of colorectal cancer (CRC) cases. In RAS signaling, activation of RAS leads to the dimerization and activation of RAF, which phosphorylates and activates MEK^4^. Activated MEK in turn phosphorylates and activates ERK^4^. Recently, chemical compounds that specifically inhibit KRAS G12C have been developed and entered the clinic^5^, but other activating KRAS mutants are still considered undruggable targets because they do not display a structure conducive to high-affinity selective small-molecule recognition^6,7^. Extensive efforts to target molecules downstream of RAS led to the identification of a variety of specific inhibitors. BRAF inhibitors are effective for BRAF V600E-mutant tumors and are in clinical use for the treatment of melanoma and a subset of lung cancer^8^. Although BRAF inhibitor monotherapy failed to exhibit a clinical benefit in CRC, the combined inhibition of BRAF, MEK and EGFR was validated in clinical trials^9,10^. Clinically effective chemotherapy for RAS-mutant cancers, unlike BRAF V600E-mutant cancers, remains to be developed due to their intrinsic resistance^11^. In RAS-mutant cancer cells, BRAF inhibitors activate downstream signaling by potentiating the interaction of CRAF and RAS^12^. MEK inhibitors transiently inhibit ERK but increase the phosphorylation of MEK, followed by rebound activation of ERK^13^. Importantly, inhibition of RAS signaling perturbed negative feedback to receptor tyrosine kinases (RTKs), which potentiated AKT signaling and bypass inhibition of RAS signaling^14,15^. Notably, the mechanisms underlying resistance to RAS signaling inhibition have been shown to be cellular context-dependent. Thus, to develop an effective therapy, investigation of the cellular response in a preclinical model that reproduces the response of tumor tissues to pharmacological intervention is highly desirable.

Patient-derived organoids (PDOs) recapitulate many of the clinical features of the original patient tumors, including the response to chemotherapeutic agents^16,17^. Familial adenomatous polyposis (FAP) is an autosomal dominant syndrome primarily caused by inherited mutations in APC gene^18^. The second hit in the APC gene causes thousands of adenomatous polyps, and the accumulation of somatic mutations, including those in KRAS and BRAF, leads to tumor progression^19^. Thus, PDOs derived from tumors in the same FAP patient have identical genetic backgrounds but distinct somatic mutations. This makes PDOs established from the same FAP patient suitable for evaluating the effects of somatic mutations in drug efficacy.

Reverse-phase protein array (RPPA) is a high-throughput proteomics technique^20^. It is an antibody-based proteomic method that can provide quantitative analysis of protein abundance and posttranscriptional modifications, including phosphorylation and glycosylation^21^. The advantage of RPPA analysis is that a large number of samples on a single slide can be quantified, which allows accurate comparative measurement of multiple samples over time after drug treatment^22^. Of note, the dynamic range of RPPA analysis is wider than that of immunoblot analysis. Here, we performed RPPA-based proteomics in combination with genomics and transcriptomics analyses to evaluate PDOs as a preclinical research platform for the development of CRC chemotherapeutic strategies.

## Results

### Establishment of PDOs from a FAP patient

**Figure 1:**
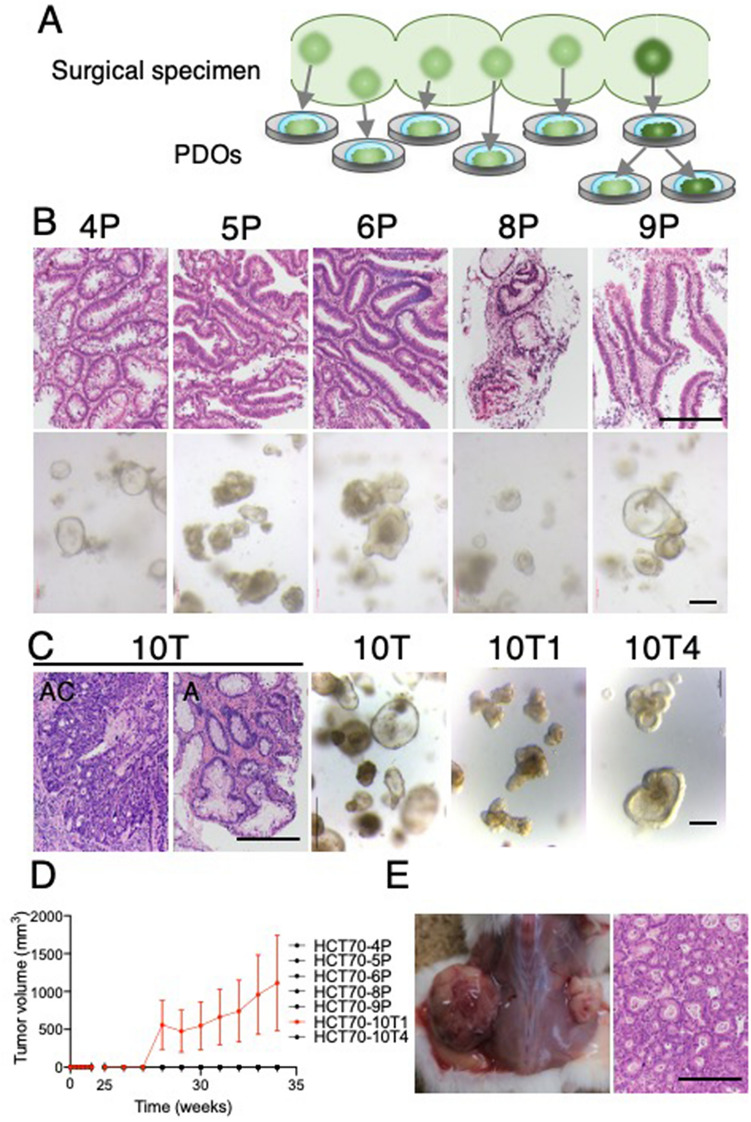
Establishment of PDOs from the FAP patient, HCT70. (**A**) Scheme showing the isolation of the independent lesions and establishment of the PDOs. Five PDOs were generated from adenomatous lesions (light green). One PDO was established from an adenocarcinomatous lesion (dark green), and two morphologically distinct subclones were established. (**B**) Histopathological analysis of original tumor specimens and PDOs from adenoma lesions. Hematoxylin and eosin (HE)-stained images of the source tumors (upper panels) and phase-contrast images of PDOs are shown. Scale bar = 200 µm. (**C**) Histopathological analysis of an adenocarcinoma lesion and the morphologies of PDOs. HE-stained images of adenocarcinoma (AC) and adenoma (A) observed in the lesion are shown. Phase-contrast images of primary PDOs (10 T) and two subclones (10T1 and 10T4) are shown. Scale bar = 200 µm. (**D**) Growth rate of subcutaneous PDO xenografts. HCT70-10T1 showed tumor formation (shown in red), while other PDOs showed no sign of engraftment (shown in black). Data are shown as the mean + /-SEM (N = 4). (**E**) Representative images of xenografted tumors and HE-stained HCT70-10T1 tumor sections. Scale bar = 200 μm.

Thousands of adenomatous lesions arise in the colorectal regions of FAP patients, and these lesions proceed to tumor development by the accumulation of somatic mutations. In this study, we established multiple PDOs from a single FAP patient (Fig. 1A). This patient, HCT70, was a 29-year-old man diagnosed with FAP in 2006 who underwent subtotal colectomy and ileorectal anastomosis. Submucosal invasive carcinoma was found by follow-up colonoscopy in 2016, and total resection of the residual rectum and ileal pouch-anal anastomosis were performed. We established five PDOs from the patient’s adenomatous lesions: HCT70-4P, -5P, -6P, -8P and -9P (P stands for polyps) (Fig. 1B). Each specimen was collected from an independent polyp that developed in the rectal region and pathologically diagnosed as tubular adenomas (Fig. 1B, upper panels). The established PDOs were morphologically indistinguishable and contained a clear space lined by a single layer of cells, indicating adenoma-derived PDOs (Fig. 1B, lower panels)^23^^.^ In addition, we established one PDO named HCT70-10 T (T stands for tumor) from a cancerous lesion that was diagnosed as moderately differentiated adenocarcinoma. Close examination revealed that this lesion contained two pathologically distinct regions of adenoma and adenocarcinoma (Fig. 1C). We noticed that among the PDOs established from this lesion, two morphologically distinct PDOs existed; they were cultured independently, resulting in HCT70-10T1 and -10T4. HCT70-10T1 contained more congested cells and had little cavity, while HCT70-10T4 exhibited a morphology similar to that of PDOs derived from adenomatous lesions. To examine the tumorigenic capacity of PDOs, they were transplanted subcutaneously into NOD/Shi-scid, IL-2Rγnull (NOG) mice. Tumor formation was observed in mice transplanted with HCT70-10T1, but no tumor formation was observed in mice transplanted with the other PDOs, demonstrating that they retained the tumorigenic potential of the original tumors (Fig. 1D). Histopathological analysis revealed that xenotransplanted tissues from HCT70-10T1 were diagnosed as adenocarcinoma, which resembled the adenocarcinoma component of the original tumor tissue (Fig. 1E).

### Mutations in APC and RAS signaling in FAP organoids

**Figure 2:**
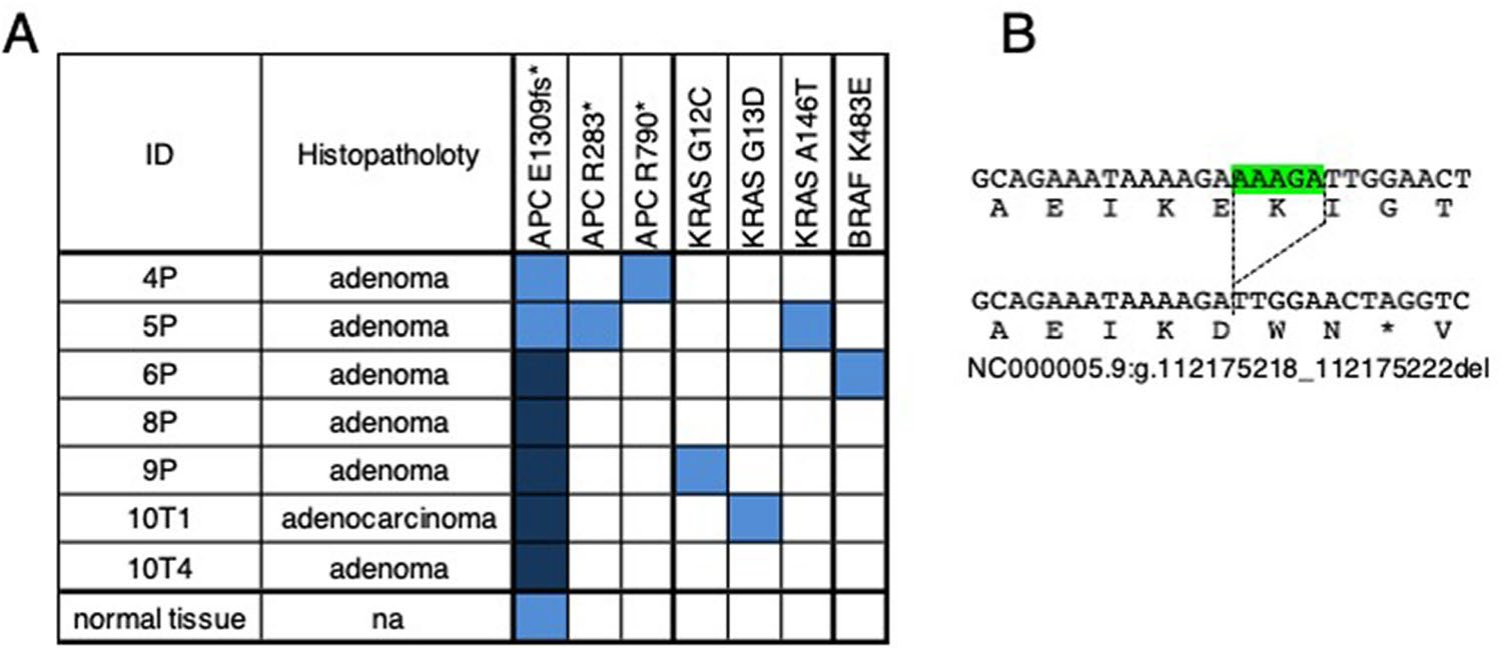
Mutations in APC, KRAS and BRAF in PDOs established from FAP. (**A**) Mutations were analyzed by targeted sequencing, and key driver mutations are shown. Other mutations identified in this analysis are shown in Supplementary Table S1. Dark blue and light blue indicate allele frequencies of more than 0.75 and between 0.25 and 0.75, respectively. Normal tissue denotes the sequence obtained from normal mucosa. (**B**) The nucleotide sequence of APC E1309fs*. The five nucleotides deletion was shown by green marks. The protein sequences caused by the mutation, NC000005.9:g112175218_112175222del, is shown in bottom.

To definitively diagnose patient HCT70, we examined mutations in the APC gene by targeted sequencing and found a five-nucleotide deletion shared by seven PDOs (Fig. 2 and Supplementary Table S1). This mutation, NC000005.9:g.112175218_112175222del, NM_000038.6:c.3927_3931delAAAGA, led to the truncated protein (NP_000029.2:p.Glu1309fsTer4). This mutation was frequently observed in the classical type of FAP, and was characterized by the presence of a large number of polyps, early-onset adenomas and a high risk of CRC^24^. This mutation was considered to be the germline mutation of this patient, because it was detected in the sequence of the normal colon mucosa of the identical patient. HCT70-6P, -8P, -9P, -10T1 and -10T4 harbored a homozygous mutation. HCT70-4P harbored additional nonsense mutations (NC_000005.10:g.112837962A > T,NM_000038.6:c2368A > T), which led to the truncated protein (NP_000029.2:p.Arg790Ter). HCT70-5P also had nonsense mutation (NC000005.10:g.112815507C > T, NM_000038.6:c.847C > T), which produced the truncated protein (NP_000029.2:p.Arg283Ter). These observations provided the evidence that patient HCT70 was affected by FAP, and the APC gene was inactivated by either loss of heterozygosity (LOH) or mutations in the second allele, consistent with the proposed model of tumor development.

Interestingly, the targeted sequence identified KRAS and BRAF mutations in multiple PDOs. HCT70-5P, -9P and -10T1 harbored the KRAS mutations A146T, G12C and G13D, respectively. HCT70-6P harbored the BRAF K483E mutation, which was classified as a class 3 mutation^25^. Since all PDOs except HCT70-10T1 were derived from adenomatous lesions and did not show tumor-forming capability, these observations suggest that the constitutive activation of KRAS signaling is an early event in the adenoma-carcinoma sequence and is insufficient for the progression to carcinoma.

### Drug sensitivities of PDOs to chemotherapeutic agents depend on somatic mutations in RAS and BRAF

**Figure 3:**
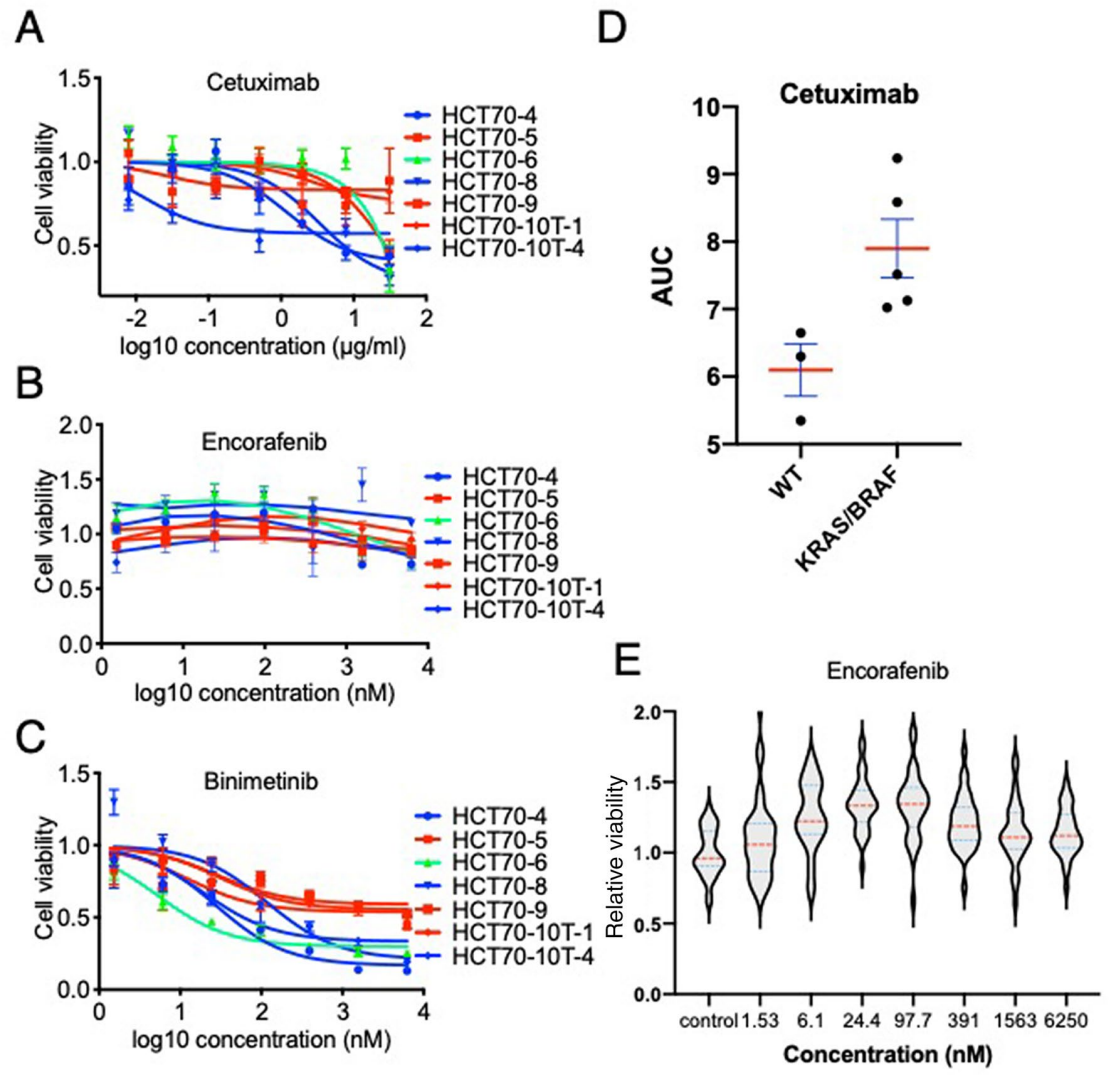
Drug responses of PDOs derived from FAP. Dose–response curves of PDOs treated with cetuximab (**A**), encorafenib (**B**) and binimetinib (**C**) at the indicated dose ranges are shown. Cell viability was measured at 72 h after drug treatment, and data were normalized by the value obtained at the beginning of drug treatment and are shown as the relative viability. Blue, green and red lines indicate PDOs with wild-type, a BRAF mutation and a KRAS mutation, respectively. Data are shown as the mean ± SEM (N = 4). (**D**) PDOs carrying an RAS signal-activating mutation were more resistant than wild-type PDOs. Each dot indicates a PDO. Red lines and blue lines indicate the mean and SEM, respectively. p = 0.0357 (Mann–Whitney test) (**E**) Encorafenib potentiated cellular viability irrespective of genetic mutations. Violin plot showing cellular viability after treatment with encorafenib at the indicated concentration. Red and blue dotted lines indicate the medians and quartiles, respectively. p < 0.0001 (one-way ANOVA).

PDOs established from HCT70 are a unique resource with which to investigate RAS signaling because they have different somatic mutations in an identical genetic background. We took advantage of this feature to explore the drug responses of CRC towards chemotherapies targeting RAS signaling. We investigated the response of PDOs to three chemotherapeutic agents clinically used for the treatment of CRC harboring the BRAF V600E mutation^9,26^: binimetinib, encorafenib and cetuximab, which inhibit MEK, BRAF and EGFR, respectively. Seven PDOs derived from the FAP patient were challenged with drugs for 72 h, and their viability was normalized by the value measured prior to drug treatment (see details in the Materials and Methods section.). As expected, PDOs harboring KRAS or BRAF mutations were more resistant to cetuximab than those without these mutations (p = 0.0357, Mann–Whitney test) (Fig. 3A,D). Encorafenib did not reduce cell viability irrespective of KRAS or BRAF mutations (Fig. 3B). ATP-competitive RAF inhibitors inhibit RAS signaling in cells with the BRAF V600E mutant but enhance signaling in those with wild-type BRAF by potentiating the interaction of CRAF with RAS^27–29^. In line with these findings, we observed increased cell viability after exposure to encorafenib (P < 0.0001, one-way ANOVA) (Fig. 3E). Notably, HCT70-6P, which harbored the BRAF K483E mutant, also responded to encorafenib, which increased cell viability. These observations demonstrated that a BRAF inhibitor potentiated further signal activation and accelerated the growth of cells with a class 3 BRAF mutation^30^. Taken together, the responses of PDOs derived from a FAP patient to cetuximab and encorafenib support the notion that they can reproduce the responses of human tumors in vivo to chemotherapeutic agents.

To explore the response of colorectal tumor cells to MEK inhibition, we examined the effect of binimetinib on cell viability and found different sensitivities depending on genotype (Fig. 3C). Three PDOs harboring KRAS mutations, HCT70-5P, -9P, and -10T1, were more resistant than wild-type PDOs. This genotype-specific response to binimetinib is in good agreement with previous clinical studies indicating that MEK inhibitors have a significant antitumor effect on BRAF-mutant tumors but a marginal effect on tumors harboring KRAS mutations^31–33^. Interestingly, PDOs with the BRAF K483E mutation (HCT70-6P) was more sensitive to binimetinib than wild-type PDOs, raising the possibility that MEK inhibition is beneficial for the treatment of CRCs with this type of BRAF mutation. These observations provide further evidence indicating that PDOs derived from this FAP patient recapitulated the drug responses of CRC tumors and are a useful resource to evaluate gene-specific responses to chemotherapeutic agents.

### RPPA revealed genotype-dependent regulation of RAS-related molecules in response to MEK inhibition

**Figure 4:**
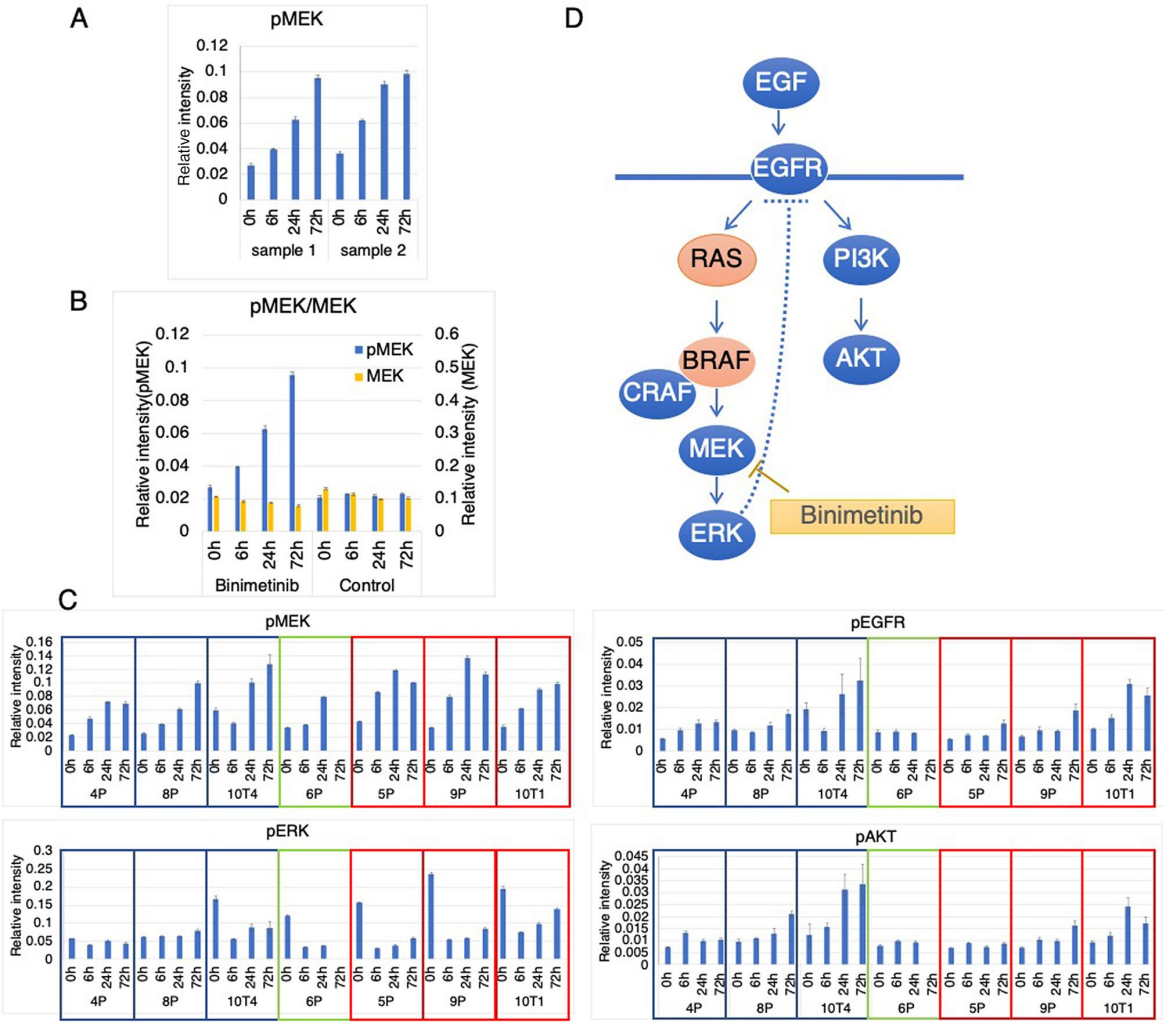
Reverse-phase protein array (RPPA) analysis of PDOs derived from FAP. (**A**) HCT70-10T1 PDOs were treated with 0.1 µM binimetinib for the indicated duration, and phosphorylated MEK (Ser217/221) was quantified by RPPA. Each spot was normalized to the tubulin intensity. Data are from two independent experiments with four spots and are shown as the mean ± SEM (N = 4). (**B**) HCT70-10T1 PDOs were treated with 0.1 µM binimetinib (binimetinib) or left untreated (control) and analyzed as described in (A). Phosphorylated MEK and total MEK are indicated by blue and orange bars, respectively. Data were obtained from four spots and are shown as the mean ± SEM (N = 4). (**C**) The indicated PDOs were treated and analyzed by RPPA as described in (A) using antibodies against phosphorylated MEK (Ser217/221), phosphorylated ERK (Thr202/Tyr204)), phosphorylated EGFR (Tyr1068) and phosphorylated AKT (Ser473). Blue, green and red frames indicate PDOs with wild-type, BRAF and KRAS mutations, respectively. Data were obtained from four spots and are shown as the mean ± SEM (N = 4). (**D**) Scheme showing RAS signaling and its inhibition by binimetinib. Arrow showed the activation of signaling molecules, while a dotted line indicates the suggested negative feedback loop downstream of ERK. The mutations analyzed in this study are shown in red.

We next focused on RAS signaling after binimetinib treatment because the responses of PDOs to binimetinib have not been fully investigated. Several mechanisms for the resistance to MEK inhibition have been suggested; these include the phosphorylation of MEK followed by the rebound activation of ERK^34^ and the suppression of negative feedback of receptor tyrosine kinases, including the ERBB family, FGFR1 and IGFR^14,15,35,36^. Notably, these resistance mechanisms were reportedly to depend on tumor type. However, these studies mostly relied on analyses of established cell lines, and their different responses may not faithfully reflect the characteristics of their original tumors. To evaluate the intracellular response of colorectal cancer to a MEK inhibitor, we analyzed RAS-mediated signaling in PDOs. We employed RPPA because it allowed highly quantitative analysis of multiple samples over a time course using a limited amount of specimen. We improved the protein extraction protocol to effectively remove Matrigel, which often hampers high-throughput proteomics analysis^37^. PDOs were treated with binimetinib, and the intensity of each target protein measured at different fluorescence wavelengths was normalized to that of tubulin in the same spots. To evaluate the reproducibility of RPPA analysis of PDOs, we repeated the experiments and obtained comparative results from two independent experiments (Fig. 4A). We confirmed that the protein levels of MEK were not affected by binimetinib treatment and that the phosphorylation level was stable without binimetinib treatment (Fig. 4B). Based on these results, we concluded that RPPA analysis was a highly reliable approach to explore the phosphorylation of PDOs in response to MEK inhibition.

To obtain the kinetics of intracellular signaling in PDOs carrying distinct somatic mutations, we analyzed the phosphorylation states of MEK, ERK, EGFR and AKT at 0, 6, 24 and 72 h after binimetinib treatment. We were not able to examine HCT70-6P after 72 h of treatment because the PDOs did not survive at this concentration of binimetinib (Fig. 3C). As previously reported in studies using two-dimensional cell lines, phosphorylation of MEK was increased by MEK inhibition in a time-dependent manner^34,38,39^. Phosphorylation of MEK was shown to be induced by the allosteric inhibition of MEK in RAS-activated cells^34^. Notably, we found that MEK phosphorylation was similarly increased in three wild-type PDOs, HCT70-4P, -8P and -10T4 (Fig. 4C). The basal level of ERK phosphorylation in KRAS- and BRAF-mutant PDOs was greater than that of WT PDOs (except HCT70-10T4) (p = 0.0346, t-test), indicating constitutive activation of RAS signaling. HCT70-10T4 exhibited a phosphorylation level of ERK compared to that of KRAS-mutant PDOs regardless of its wild-type KRAS gene, suggesting KRAS-independent activation of ERK. At 6 h after treatment, ERK phosphorylation in PDOs with high basal activity was reduced ranging from 20% (HCT70-5P) to 38% (HCT70-10T01) and then restored ranging from 36% (HCT70-5P) to 71% (HCT70-10T1) at 72 h after treatment. Notably, ERK phosphorylation was only marginally affected, if at all, in the two wild-type PDOs HCT70-4P and -8P, and no significant rebound was detected. Taken together, these observations demonstrated that the activating mutation of KRAS potentiated the basal level of ERK phosphorylation. MEK inhibition transiently reduced ERK activity, followed by rebound activation. These responses are consistent with previous reports and may explain the resistance of PDOs to binimetinib. However, our data suggested that rebound activation was not sufficient for resistance because HCT70-10T4 exhibited a response similar to that of KRAS-mutant PDOs but was sensitive to binimetinib.

We next analyzed EGFR because it is a potential therapeutic target for CRC. Mechanistically, MEK inhibition activates EGFR by perturbing a feedback inhibition loop (Fig. 4D, broken line). Consistent with this model, we observed increased phosphorylation of EGFR in a time-dependent manner after binimetinib treatment. Furthermore, AKT, a downstream target of EGFR, was similarly phosphorylated. These observations demonstrated that in CRC, MEK inhibition leads to functional activation of the EGFR/AKT axis. Interestingly, EGFR phosphorylation was observed in HCT70-4P and -8P, which did not have KRAS or BRAF mutations, suggesting that constitutive activation of RAS signaling is dispensable for activation of the EGFR-AKT axis in response to binimetinib treatment.

**Figure 5:**
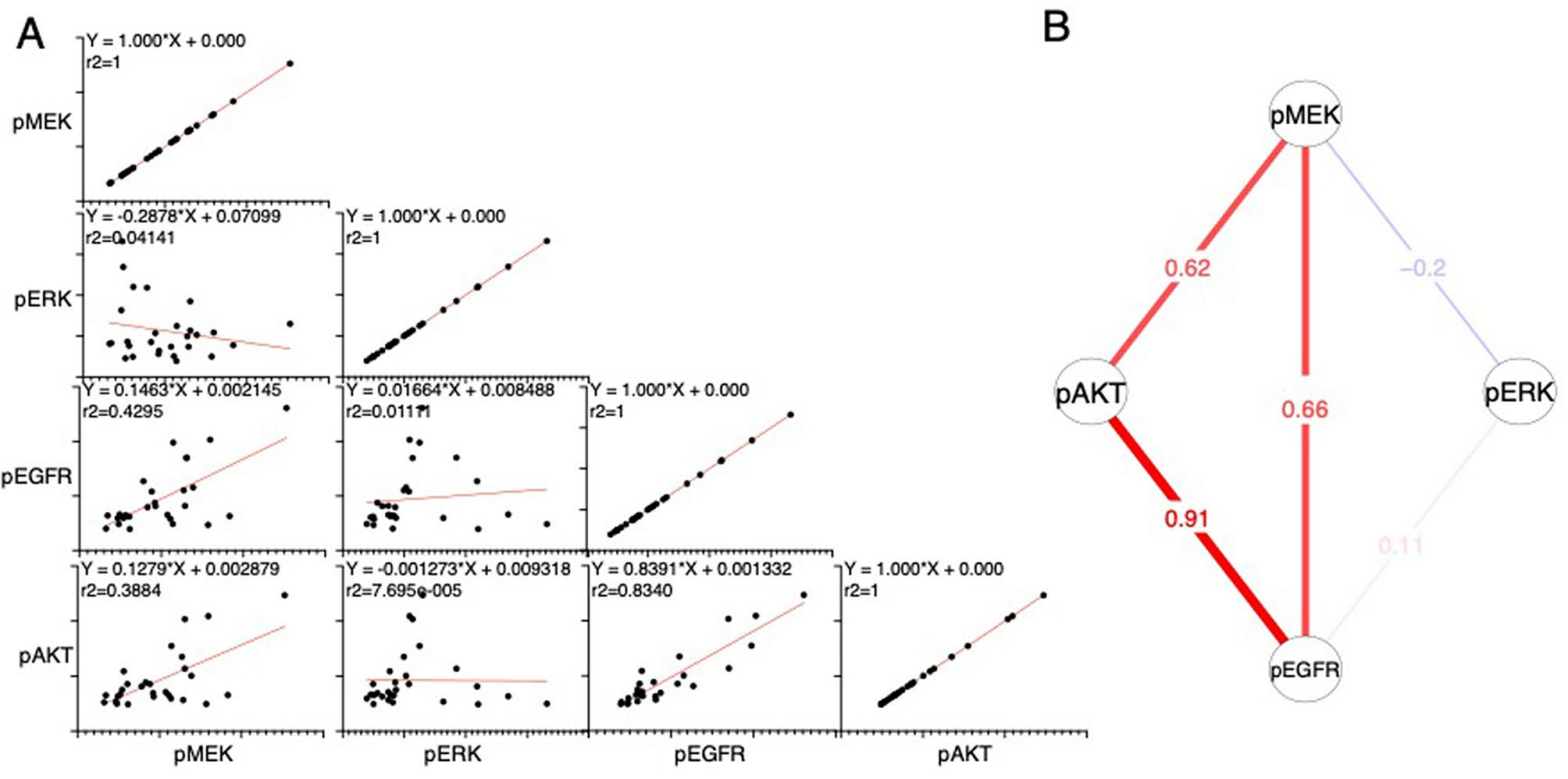
Correlation of phosphorylated proteins in response to binimetinib treatment. (**A**) Simple linear regression curves for the indicated variables are shown. The equation and R squared value for each graph are indicated. (**B**) Pearson correlations of the indicated variables were determined using RPPA data, and positive and negative correlations are shown in red and blue, respectively.

To gain further insights into the link among phosphorylation events after MEK inhibition, we performed linear regression analysis (Fig. 5A). We found a strong relationship between pEGFR and pAKT (r^2^ = 0.834). Consistently, their phosphorylation was tightly correlated in Pearson correlation analysis (r = 0.9132, p < 0.0001) (Fig. 5B), suggesting strong regulation of pAKT by EGFR. A correlation between pMEK and pERK was not observed by either linear regression analysis or Pearson correlation. Interestingly, pEGFR and pAKT were shown to be correlated with pMEK rather than pERK. These observations suggested that, in addition to perturbation of the feedback loop downstream of ERK, the EGFR-AKT axis was activated by additional mechanisms downstream of MEK, although the phosphorylation of MEK may not indicate its kinase activity because binimetinib is an allosteric inhibitor of MEK.

### Comprehensive genome analysis revealed the distinct somatic mutation profiles of PDOs

**Figure 6:**
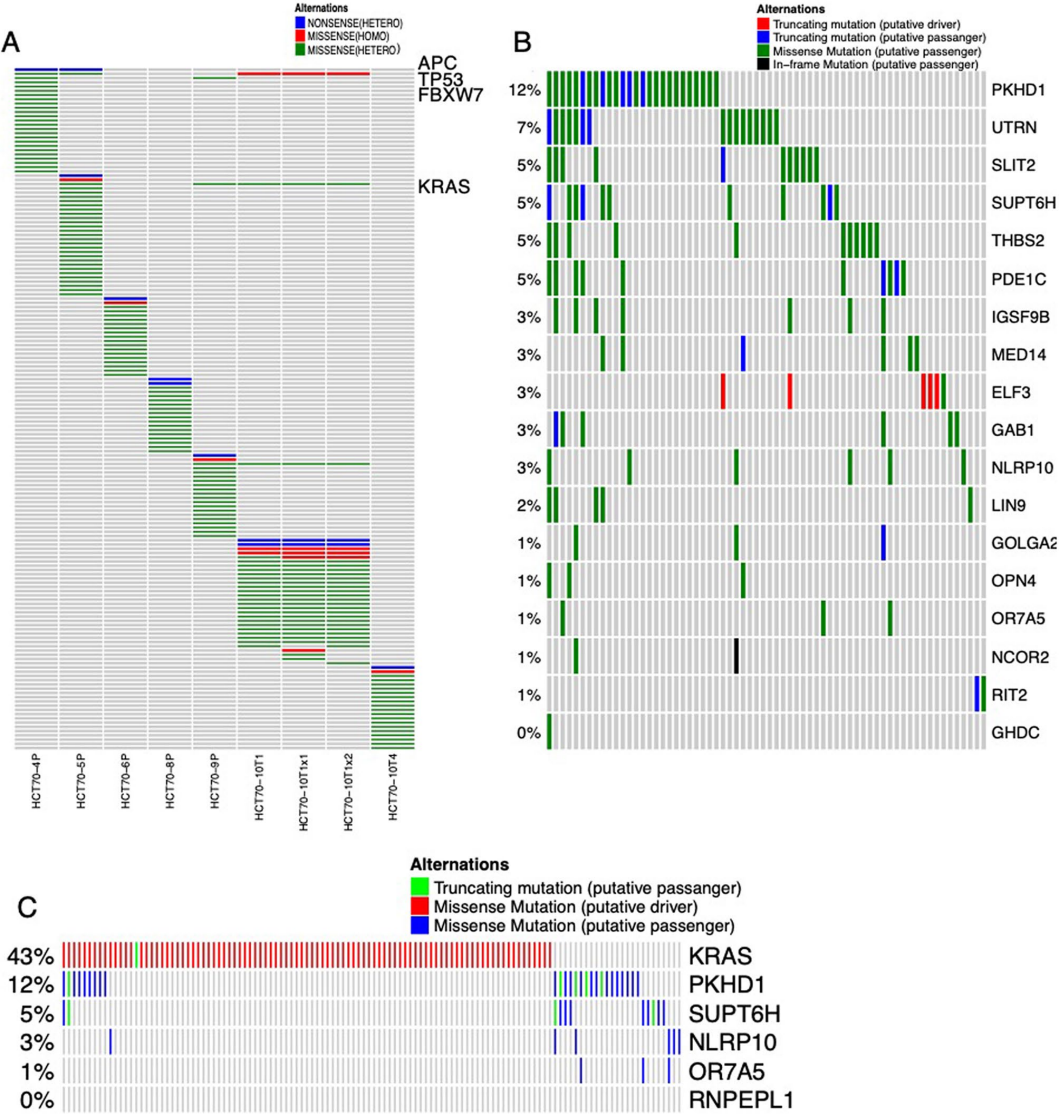
Somatic mutations in PDOs derived from a FAP patient. (**A**) Mutations determined by exome sequencing are shown. Recurrent mutations in multiple PDOs, APC, TP53, FBXW7 and KRAS, are indicated. The full list of genes is shown in Supplementary Fig. S2 and Supplementary Table S5. (**B**) Mutation frequencies of genes in colorectal adenocarcinoma tissues in TCGA data set (Firehose Legacy, cBioportal). Genes in which mutations are identified in HCT70-10T4 are shown. Of 21 genes, no mutations are found in three genes and 18 genes are listed in this panel. (**C**) Mutually exclusivity of mutated genes found in HCT70-10T4 with KRAS mutations. 18 genes listed in (**B**) are analyzed in the same data set, and top five genes exhibiting mutual exclusive tendency are shown. Detailed description is presented in Supplementary Table S6.

Since resistance to MEK inhibition was attributed to intrinsic cellular properties, we next characterized the genomes of PDOs. RPPA demonstrated that PDOs exhibiting high basal ERK phosphorylation harbored activating mutations in RAS signaling, except HCT70-10T4, which had a high basal level of ERK phosphorylation regardless of the presence of the wild-type KRAS gene. Thus, we carried out exome sequencing to obtain a detailed somatic mutation profile (Fig. 6A, Supplementary Fig. S2, Supplementary Table S5). We included two PDOs, HCT70-10T1 × 1 and HCT70-10T1 × 2, which were established from mouse subcutaneous graft tissue from HCT70-10T1, and found that these graft-derived PDOs have similar mutation profiles to HCT70-10T1. Four potential driver mutations of CRC, APC, TP53, FBXW7 and KRAS, were recurrent in our cohort. Interestingly, most of the other mutations were specific to each PDO. These observations suggested that each PDO acquired unique somatic mutations, which could potentially have conferred the unique biological features. Hence, we asked whether 21 somatic mutations found in HCT70-10T4 could contribute to the ERK phosphorylation. To examine the possible interaction with RAS signaling, we investigated the mutation frequency and their mutual exclusivity with KRAS mutations in human colorectal cancer using the mutation profile of TCGA database. As shown in Fig. 6B, 18 mutations were detected in this cohort, and no mutations were detected in three genes. Five genes, including PKHD1, SUPT6H, NLRP10, OR7A5 and RNPEPL1, showed a tendency to be mutually exclusive with KRAS (Fig. 6C, Supplementary Table S6). These observations raised the possible contributions of these genes to high basal ERK phosphorylation independent of KRAS mutation. Further comprehensive analysis including mutations in untranscribed regions and chromosome aberrations, could account for the high basal ERK phosphorylation in PDOs without mutant RAS.

### MYC-mediated transcription and the interferon response are closely related to sensitivity to MEK inhibition

**Figure 7:**
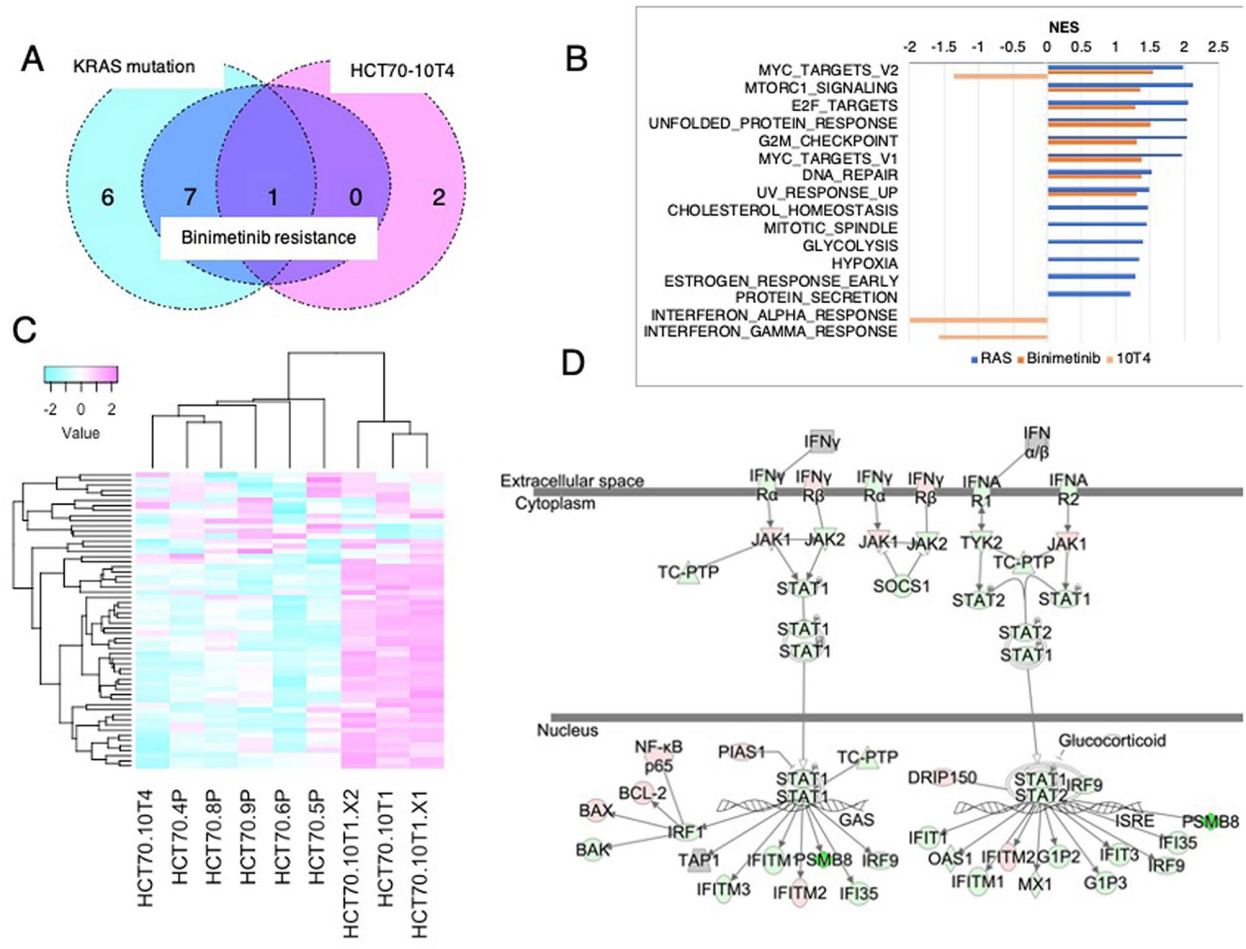
Expression profiles of PDOs derived from FPA. (**A**, **B**) Gene set enrichment analysis (GSEA) revealed 8, 14 and 3 gene sets significantly correlated with binimetinib resistance, KRAS mutation and HCT70-10T4 PDOs, respectively (NES > 1.2). The PDOs sets used in each analysis are described in materials and methods. A Venn diagram showing the number of gene sets common to PDOs sets and specific to PDO sets (**A**) and a graph indicating the NES of the enriched gene sets (**B**) are shown. (**C**) Heatmaps of the mRNA expression of genes in the MYC targets V2 gene set are shown. Each row was transformed using the Z-score. The color represents mRNA expression levels scaled across samples. Genes and samples were hierarchically clustered using Pearson correlation. The full list of genes is shown in Supplementary Fig. S3. (**D**) Ingenuity pathway analysis (IPA) of gene expression in HCT70-10T4. Differentially expressed genes in HCT70-10T4 PDOs are overlaid on the interferon signaling pathway. Red and green indicate higher and lower expression in HCT70-10T4 PDOs compared to wild-type PDOs, respectively.

We next analyzed the expression profiles of FAP PDOs. Gene set enrichment analysis (GSEA) of RNA-seq data identified eight gene sets significantly upregulated in binimetinib-resistant PDOs compared to binimetinib-sensitive PDOs (Fig. 7A,B, Supplementary Table S2). These genes were among those upregulated in PDOs harboring KRAS mutations, supporting the notion that KRAS mutations are the major somatic mutations that are related to resistance to MEK inhibition (Supplementary Table S3). We identified three gene sets by the comparison of HCT70-10T4 with other wild-type PDOs (Supplementary Table S4). Among them, MYC targets V2, which contains key downstream targets of RAS signaling, was also identified as a gene set related to binimetinib resistance and KRAS mutation (Fig. 7A, B).

Unbiased analysis of the MYC targets V2 gene set revealed clusters correlated with the tumor histopathology, and adenocarcinoma-derived PDOs exhibited the high expression of distinct genes from adenoma-derived PDOs related to cellular proliferation, including CDK4, PLK1 and 4, MCM4, and MYC (Fig. 7C, Supplementary Fig. S3). Three wild-type PDOs formed a cluster, but HCT70-4P and HCT70-8P were closer to each other than HCT70-10T4. Overall, the clusters of gene sets in MYC targets V2 in the PDOs were more correlated with binimetinib sensitivity than with ERK phosphorylation.

Interestingly, we identified interferon alpha and gamma response genes as gene sets downregulated in HCT70-10T4 (Fig. 7B). The downregulation of STAT1/2 and their target genes in these PDOs was also evident by Ingenuity pathway analysis (IPA) (Fig. 7D). These gene sets were reported to be highly related to the resistance of CRC to MEK inhibition, and STAT1-mediated transcription was shown to be higher in resistant PDOs than in sensitive PDOs^40,41^. These observations support the notion that a reduced interferon response may confer HCT70-10T4 sensitivity to MEK inhibition irrespective of its high level of ERK phosphorylation.

## Discussion

In this study, we analyzed seven PDOs from a single FAP patient. In our previous study, 42 PDOs were established from five FAP patients, but only four KRAS-mutant PDOs from four different patients were obtained, and no BRAF-mutant PDOs were obtained^40^. In contrast, three KRAS-mutant PDOs and one BRAF-mutant PDOs derived from the same patient were included in this study, which allowed direct comparative analysis of somatic mutations because their genetic backgrounds were identical. We focused on the impact of somatic mutations on the responses to chemotherapeutic agents because mutations found in PDOs naturally occur in CRC patients under physiological conditions, and thus, PDOs can reproduce the response of tumor tissues more precisely than genetically engineered cell lines.

In the clinical setting, pharmacological interventions have often failed to demonstrate a significant benefit in patients with RAS mutations. A large body of work has provided mechanical insights into drug resistance, which often is dependent on the cellular context, including the tumor type. We addressed this issue by taking advantage of PDOs that maintain the cellular and biological features of the original cancer tissue. We showed that PDOs harboring KRAS mutations were resistant to cetuximab. They showed accelerated growth in response to encorafenib treatment, which is consistent with the paradoxical activation of ERK by BRAF inhibition. These results supported the notion that these PDOs faithfully reproduced clinical responses and indicated their value as an in vitro model for CRC. Interestingly, PDOs carrying the BRAF K483E mutation were resistant to cetuximab but hypersensitive to binimetinib. Mutant BRAF proteins normally function as either activated monomers or constitutive dimers with wild-type BRAF and CRAF^42,43^. Dela Cruz et al. reported that BRAF K483E expression potentiated ERK activation although it harbors a catalytically dead kinase domain^30^. In line with this report, we observed elevated basal ERK activity. These results provide further evidence that BRAF K483E is an activating mutation of RAS signaling with effects likely mediated through allosteric activation of its dimer partner. Recent clinical research demonstrated that EGFR inhibition showed clinical benefit in a subset of CRC patients harboring class 3 BRAF mutations^44^, but its underlying mechanisms are elusive. Further analysis of PDOs may deepen our understanding for the development of an effective therapeutic strategy.

To delineate RAS signaling in response to MEK inhibition, we employed RPPA, a sensitive, high-throughput, functional proteomic technology that offers many of the advantages needed to quantify proteins in the normal and tumor tissues of CRC patients^45,46^. In this study, we carried out a highly quantitative analysis of phosphorylation using small amount of protein samples, which allowed us to sensitively and accurately compare signaling molecules among multiple PDOs. We observed increased phosphorylation of MEK after binimetinib treatment, which is well documented as a response of KRAS-mutant cells to allosteric inhibition of MEK. We noticed that this response in wild-type PDOs was comparable to that in PDOs harboring an activating mutation in KRAS. Nevertheless, their ERK phosphorylation responses were different. As previously reported, we observed rebound activation of ERK in KRAS-mutant PDOs^28,34^. In contrast, MEK inhibition led to an only marginal reduction, if any, in ERK phosphorylation, and no significant rebound phosphorylation was detected in wild-type PDOs. Interestingly, phosphorylation of EGFR and AKT was also observed in wild-type PDOs. These observations suggested that enhanced activation of EGFR alone does not explain the resistance of RAS-mutant PDOs to MEK inhibition. Notably, phosphorylation of EGFR and AKT was more correlated with MEK phosphorylation than phosphorylation of ERK (Fig. 5B). These correlations may predict that in addition to perturbing the ERK-dependent negative feedback of EGFR, the EGFR-AKT axis is activated through mechanisms that are correlated with MEK phosphorylation, although phosphorylation of MEK and its kinase activity in the presence of binimetinib need to be evaluated. Overall, these observations demonstrated that RPPA analysis of PDOs provides an opportunity to gain novel insights into RAS signaling in response to pharmacological intervention.

In this study, we showed the RPPA analysis of PDOs is an effective approach to understand the cellular signaling in response to the chemotherapeutic agent. This analysis also uncovered the elevated basal level of ERK phosphorylation in HCT70-10T4. In the transcriptome analysis, we found MYC_target V2 gene sets, which represented downstream targets of the RAS signaling, was downregulated in HCT70-10T4, which may explain its sensitivity to binimetinib despite of the high basal MEK phosphorylation. Unbiased clustering using this gene sets demonstrated HCT70-10T4 was in close proximity with HCT70-4P and -8P, reflecting their response to binimetinib (Fig. 6C). Additionally, we found two gene sets, interferon alpha and gamma response, were negatively correlated to HCT70-10T4, consistent with the previous studies demonstrating their higher activity conferred the resistance to MEK inhibitors. Taken together, this study demonstrated that the combined analyses of proteome, transcriptome and genome helped us to deepen our understanding in the cellular responses to chemotherapeutic agents.

Since the discovery of RAS mutations in a variety of human cancers, extensive efforts have been made to develop selective inhibitors of RAS signaling. However, many agents have failed to demonstrate a significant benefit in patients with RAS mutations, which is due to intrinsic resistance. A large number of studies carried out to develop therapeutic strategies have highlighted that combination with other chemotherapeutic agents relieves resistance. Although we used a limited number of PDOs and analyzed a subset of RAS signaling molecules, this study demonstrated the feasibility of PDOs as a preclinical model of therapeutic development. We also showed the validity of RPPA for comprehensive analysis of protein phosphorylation. In conclusion, RPPA analysis of PDOs in combination with genome and transcriptome profiling provides an excellent opportunity to gain insights into RAS signaling and to develop chemotherapeutic strategies to overcome resistance to molecular targeting agents.

## Materials and methods

### Establishment of colorectal tumor organoids

All samples and methods used in this study were approved by the Ethics and Medical Research Committee of the Japanese Foundation for Cancer Institute (approval number: 2013-1105). Clinical samples used for organoid establishment and biological analyses were obtained from a FAP patient at the Cancer Institute Hospital that gave informed consent after the approval of the Ethics and Medical Research Committee of the Japanese Foundation for Cancer Institute. We confirm that all research was performed in accordance with relevant guidelines and regulations.

Colorectal tumor specimens were collected by surgical resection, and histological diagnosis was determined by an experienced pathologist. PDOs were established as previously reported^23^. Briefly, surgical specimens were washed with phosphate-buffered saline (PBS) and minced into 1 mm^3^ fragments using surgical scissors. The fragments were digested with digestion buffer (Dulbecco’s modified Eagle’s medium (DMEM), fetal bovine serum, penicillin/streptomycin, collagenase and dispase) at 37 °C for 60 min. Isolated tumor cells were embedded in Matrigel droplets and overlaid with culture medium prepared as previously described^23^. Advanced DMEM/F12 medium was supplemented with penicillin/streptomycin, 10 mM HEPES, 2 mM GlutaMAX, 1 × B27 (Thermo Fisher Scientific), 10 nM gastrin I (Sigma), and 1 mM N-acetylcysteine (Wako, Japan) to prepare basal culture medium. Complete medium was prepared by supplementing the basal culture medium with the following niche factors: 50 ng/ml mouse recombinant EGF (Thermo Fisher Scientific), 100 ng/ml mouse recombinant noggin (Peprotech), and 1 mg/ml human recombinant R-Spondin 1 (R&D). The media were changed every 2 or 3 days.

### Targeted sequencing and exome sequencing

Genomic DNA was extracted with the QIAamp DNA Mini kit (Qiagen), and the quality was validated using the Qubit system (Thermo Fisher Scientific). Targeted sequencing of 69 genes that were recurrently mutated in colorectal adenocarcinoma was performed^40^. Custom probe panels were designed using the SureDesign tool (Agilent Technologies), and a library was constructed using the HaloPlex Target Enrichment System Kit (Agilent Technologies). Sequencing was performed with 150 bp paired ends by a MiSeq system (Illumina) using a MiSeq Reagent Kit (Illumina). The fastq files were analyzed by SureCall v3.5 (Agilent) using the default settings. The raw sequence reads were aligned on GRCh37, and variants were called using SNPPET. The mean sequencing depth for the targeted sequencing regions was approximately 500×. To detect mutations, raw variants were filtered by removing those registered in the Japanese SNP database (Human Genetic Variation Database, release v2.30) with a frequency of > 0.01. Somatic mutations in the PDO samples were identified through comparison with frozen colon mucosa tissue.

Exome sequencing was performed by DNA Link, Inc. (Seoul, Korea) through GeneBay, Inc. (Yokohama, Japan). Following the manufacturer’s instructions, the genomic DNA was fragmented and used to prepare paired-end libraries using the SureSelect Human All Exon V6 kit (Agilent). Sequencing was performed using the Illumina HiSeq2500 system. The fastq files were trimmed using Cutadapt and mapped onto the human reference genome GRCh37 (hg19) using BWA v0.7.17. The mapped reads were processed with the Genome Analysis Toolkit (GATK, v4.0.4.0) following best practices workflows from GATK. Germline single-nucleotide variations (SNVs), insertions, and deletions in the normal samples were detected using the GATK HaplotypeCaller command, and somatic SNVs, insertions and deletions in PDOs were detected using GATK Mutect2. The mean sequencing depth of the targeted sequencing regions was approximately 100 ×. Variants were quality-filtered by using the GATK variantFiltration command for the germline variants and FilterMutectCalls command for the somatic variants. The variants were then annotated using the SnpEff v4.3q program with COSMIC database v71, and common variants were registered in dbSNP build 150.

### RNA sequencing

Total RNA was extracted with an RNeasy mini kit (Qiagen) and quantified using 2100 Expert Bioanalyzer software (Agilent). RNAseq analysis was performed by DNA Link, Inc. (Seoul, Korea) through GeneBay, Inc. (Yokohama, Japan). Following the manufacturer’s instructions, total RNA with RIN > 7 was used to generate a barcode-labeled library using the Illumina TruSeq Stranded mRNA LT Sample prep kit (Illumina). Sequencing of 150 bp paired-end reads was performed on the Illumina NovaSeq6000 system (Illumina). Splice mapping of the sequence reads to the GRCh37 genome was performed with the DRAGEN Bio-IT Processor (Edico Genome). On average, RNAseq yielded 5 × 10^7^ mapped reads (99%). Read-covered regions per geneID were counted and normalized using the cuffdiff program. GSEA for comparisons of binimetinib-resistant (HCT70-5P, -9P, -10T1, -10T1 × 1 and -10T1 × 2) vs binimetinib-sensitive PDOs (HCT70-4P, -6P, -8P and -10T4), KRAS-mutant (HCT70-5P, -9P, -10T1, -10T1 × 1, -10Tx2) vs wild-type PDOs (HCT70-4P, -8P), and HCT70-10T4 vs wild-type PDOs (HCT70-4P, -8P) was performed with the GSEA Java application downloaded from the Broad Institute with the default parameters.

### PDO viability assay

The drug sensitivities of PDOs to three clinically used chemotherapeutic agents, binimetinib, encorafenib and cetuximab, were tested. Stock solutions of binimetinib and encorafenib at 10 mM were prepared by dissolving in DMSO. Stock solutions were prepared by diluting compounds in DMEM to the following concentration ranges: binimetinib and encorafenib, 0 to 6.25 μM; cetuximab, 0 to 200 μM. Organoids were digested in TrypLE Express (Thermo Fisher Scientific) supplemented with 10 μmol/L Y27632 at 37 °C for 15 min with pipetting every 5 min. The cells were suspended in basal medium, and clumps were removed by passing the suspension through a 40 μm cell strainer. The suspension was centrifuged at 1000 × g for 5 min, and the cells were resuspended in basal medium. Cells were counted and adjusted to 1.5 × 10^5 ^cells/mL in 50% Matrigel in ENR medium. Two microliters of cell suspension was dispensed into each well of clear-bottom, white-wall 384-well microplates (Greiner). PDOs were cultured for three days to allow the formation of organoids and then treated with drugs at the indicated doses for three days. We generated 8-step, four-fold drug matrices in technical quadruples. Cell viability was measured using a Real time-Glo MT Cell Viability Assay (Promega). Readouts were obtained at day 0 and at day 3 using a Mithras LB 940 luminometer (Berthold Technologies). The data were analyzed using GraphPad Prism 7 software (GraphPad) and R.

### Reverse phase protein array

Details of the RPPA methodology and the validation of RPPA have been previously described^47^. PDOs were treated with binimetinib (0.1 μM) or left untreated and collected at 6 h, 24 h and 72 h after treatment. We noticed that the extensive removal of Matrigel was crucial for the accurate antibody-based quantification of phosphorylated proteins. For this purpose, PDOs were washed three times with PBS and mixed with 1 ml of Cell Recovery Solution. The mixture was incubated on ice for 1 h with occasional pipetting, washed three times with PBS, and dissolved in T-PER protein extraction reagent (Thermo Fisher Scientific) supplemented with inhibitors (100 mm NaF, 1 mm Na_3_VO_4_, 10 mm NaPPi, 1 mm EDTA, PhosSTOP; Sigma-Aldrich) and protease inhibitor cocktail (1.04 mM AEBSF, 800 nM aprotinin, 40 μM bestatin, 14 μM E-64, 20 μm leupeptin, and 15 μM pepstatin A (Sigma–Aldrich)). Cell lysates were centrifuged at 15,000 *g* at 4 °C for 20 min, and the supernatant was subjected to the following analysis. The protein concentration was adjusted to 1.0 mg/ml according to the Bradford protein assay (Bio-Rad), and printed on nitrocellulose-coated slides in four replicates (Grace Bio-Labs) using an Aushon Biosystems 2470 arrayer (Burlington). The following antibodies were used as probes: anti-phosphorylated MEK (Ser217/221) (CST9154), anti-phosphorylated ERK (Thr202/Tyr204) (CST4370), anti-phosphorylated EGFR (Tyr1068) (CST3777), anti-phosphorylated AKT (Ser473) (CST4060), and anti-MEK (CST8727). The specificity of the probe antibody was validated by immunoblot analysis (Supplementary Fig. S1). All probe antibodies were visualized using anti-rabbit antibody conjugated to infrared dyes, IRDye 680RD (LI-COR, Biosciences). Anti-tubulin (clone 64, Sigma) was used as a control probe for each spot, followed by anti-mouse antibody conjugated to IRDye 800CW (LI-COR biosciences). The signal intensity of each spot was detected using an Odyssey scanner (LI-COR Biosciences), and the amount of phosphorylated protein was normalized to tubulin.

### Statistical analysis

Drug responses and the results of linear regression model analysis and Pearson correlation analysis of phosphorylated proteins were analyzed using GraphPad Prism (GraphPad Software, Inc.) and R packages. Data for each experimental PDO are expressed as the mean ± SEM. Confidence intervals of 95% or better were considered significant. For further statistical details, refer to each figure legend.

## Supplementary information

Supplementary file 1.

Supplementary file 2.

## Data Availability

The datasets generated in this study are available from the corresponding author on reasonable request. RNA-seq data have been deposited in DDBJ Sequence Read Archive (DRA) through accession number PRJDB10442.
