# Supplementary material for: Evaluation of the RAS signaling network in response to MEK inhibition using organoids derived from a familial adenomatous polyposis patient
Source: Sci Rep. 2020 Oct 15;10:17455. doi: 10.1038/s41598-020-74530-x (PMC7567075; doi:10.1038/s41598-020-74530-x)
Supplement: Supplementary file 1 [file 41598_2020_74530_MOESM1_ESM.docx]

**Supplementary Information**

**Evaluation of the RAS signaling network in response to MEK inhibition using organoids derived from a familial adenomatous polyposis patient.**

Hiroki Osumi^1,2,3^, Atsushi Muroi^4^, Mizuho Sakahara^3^, Hiroshi Kawachi^5^, Takuya Okamoto^1^, Yasuko Natume^1^, Hitomi Yamanaka^1^, Hiroshi Takano^1^, Daisuke Kusama^1^, Eiji Shinozaki^2^, Akira Ooki^2^, Kensei Yamaguchi^2^, Masashi Ueno^6^, Kengo Takeuchi^5^, Tetsuo Noda^3^, Satoshi Nagayama^6^, Naohiko Koshikawa^4^, and Ryoji Yao^1^

^1^Department of Cell Biology, Cancer Institute, Japanese Foundation for Cancer Research, Tokyo, Japan

^2^Department of Gastroenterology, Cancer Institute Hospital, Japanese Foundation for Cancer Research, Tokyo, Japan

^3^Director’s office, Cancer Institute, Japanese Foundation for Cancer Research, Tokyo, Japan

^4^Division of Cancer Cell Research, Kanagawa Cancer Center Research Institute, Yokohama, Japan

^5^ Department of Pathology, Cancer Institute Hospital, Division of Pathology, Cancer Institute, Japanese Foundation for Cancer Research, Tokyo, Japan

^6^Department of Gastroenterological surgery, Cancer Institute Hospital, Japanese Foundation for Cancer Research, Tokyo, Japan

**Supplementary Fig. 1. Validation of probe antibodies used for RPPA analysis.** Cells were cultured in a serum-starved condition for 24 hours. A431 was treated with 100ng/ml of EGF for 15 minutes before preparing cell extract (A431+EGF). Other cells were left untreated. 10µg of protein extract were separated on SDS-PAGE and blotted with indicated antibodies.

**Supplementary Fig. 2. Somatic mutations in PDOs derived from a FAP patient.** Mutations determined by whole exome sequencing are shown. The genes were shown in right.

**Supplementary Fig. 3.** Heatmaps of the mRNA expression of genes in the MYC targets V2 gene set are shown. Gene names were shown in right. Each row was transformed using the Z-score. The color represents mRNA expression levels scaled across samples. Genes and samples were hierarchically clustered using Pearson correlation.
